# Some Medical Considerations of Sports and Games

**Published:** 1939

**Authors:** J. E. Lovelock

**Affiliations:** Olympic Champion and World Record Holder


### SOME MEDICAL CONSIDERATIONS OF SPORTS AND GAMES.

BY

J. E. Lovelock, B.M., B.Ch.,

*Olympic Champion and World Record Holder.*

---

In this short article I wish to emphasize a few points in the application of medicine to sport and games, points which are or should be well known to every doctor, but which tend to be overlooked because disease is more often in the mind of the doctor than is health.

The disinclination of clumsy and inexpert children to take part in organized physical exercises, games and athletic sports is one which every person interested in young people's health and welfare is constantly meeting. Of recent years I have found it in all grades of society and among all ages of children, whether it is among schoolchildren in the various types of schools, State or Public, or among the members of boys' clubs and similar institutions, particularly in the poorer districts of our large cities. This self-consciousness can largely be dispelled by an enthusiastic sports master or games adviser, but it is mainly to the medical man that the more careful of these physical fitness experts will appeal for help and advice. It is the family doctor, the school medical officer, and the medical adviser to the club, who can so often persuade the child or young adult, poorly blessed by nature as far as his physique is concerned, that by steady effort he can make his perhaps malformed body into a comparatively normal and healthily active one. But in order to do that adequately the medical man must have some good understanding of the essentials of physical movement, of its place in the raising of the general bodily health, and above all, must be prepared to treat the children, not as patients, with attention focused on disease or potential disease, but as living thinking human beings aiming at getting the best out of their bodies and their minds.

It is important to lay stress from the beginning on the obvious and essential association between the mental and the physical sides of sport. All forms of athletic sports and games must appeal to the mind and to the imagination of the player, and above all in the case of the less expert child, for if he or she can be sufficiently interested to forget self-consciousness, in time self-confidence will lead to steady improvement in physique.

The doctor can do a great deal to foster self-confidence by assuring the youngster of his ability to take part in sports and games which are suited to his capacity. The doctor is the best judge of what sports and games are suitable: it then rests with the enthusiasm of the sports master or games coach to carry on aided by periodic encouragement and support from the doctor.

When we come to the more directly physical medical side, I want particularly to emphasize the importance of careful routine examination of all young people taking part in sport, so that should any abnormality be found its significance can be assessed and advice given as to the type and degree of strenuousness of sport suitable for each case. Periodic examinations should certainly be made every term of schoolchildren; others undertaking strenuous competitive sport should have similar routine examinations made at least twice a year. Then attention can be given to various minor troubles such as diseased tonsils or bad teeth, which in themselves are relatively harmless but can lead to a serious undermining of the general health. Gross postural defects and skeletal deformities come into the domain of the doctor, whose advice is indispensable to games masters and coaches.

This brings me to the question of general health in sport. Too many people who wish to take part in games will not realize that good general health is the basis of success in any game. They tend to forget that before any one part of the body can put out its best performance the whole body must have its threshold of general health raised. It is the duty of the doctor to explain, particularly to the younger participants, the essential wisdom of playing different forms of games and sports in order to develop the whole body evenly. Most schoolchildren are rightly discouraged by their games masters from too early attempts at specialization: but in these days there is a tendency amongst the young to concentrate only on one or two sports to the exclusion of all others and to the detriment of their own general development. It should be the aim of games to develop personality and character, poise and self-confidence, symmetrical and well-controlled bodies as well as soundly balanced minds. The doctor is frequently consulted about the details of general fitness—such as the amount and type of exercise, diet, food and drink, alcohol and tobacco and their place in sport, massage and baths, and a host of similar points. He must have his own ideas clearly defined, and must be a good enough physiologist to explain to both participants and coaches the scientific reasons for his advice, for in these days the young are very critical and by no means ready to accept advice blindly. He must be able to explain to them just why exercise in moderation improves the health, why muscles increase in bulk and even more in utility with use, why alcohol, though apparently a stimulant, is in fact a physical depressant, like tobacco. He should be able to advise on the much over-worked subject of diet, of which many people make an unnecessary fetish : simple health rules on food should include the need for fruit and vegetables, milk and butter, the importance of small, more frequent meals well masticated taken at regular times of the day, and never just before or just after violent exercise. He should emphasize the essential importance of sleep in order that the body tissues, fatigued by exercise during the day's activities, may have an opportunity to recover, and the growing body the best chance to fill out. This is a subject to which the average person does not give enough attention. Eight hours' sleep a night is a good average, but young, growing people need much more. Regular hours of sleep are as necessary as regular exercise and regular meal times, while the importance of a quiet, well-ventilated room in the production of restful sleep is only partly realized by most young people. Hosts of other general considerations are certainly necessary for the doctor who realizes that his work among young people and especially in sports and games is mainly that of a practitioner of preventive medicine.

Many of the deformities, the injuries, or even just the disappointments and failures which ultimately come his way could be prevented by careful periodic examination, instruction and prophylactic treatment.

To quote Sir E. Kaye Le Fleming in his foreword to Dr. Griffin's *The Scientific Basis of Physical Education* : " The G.M.C. rightly insists on the teaching of medical science from the preventive aspect, yet the amount of attention given to this exhortation will, in the opinion of not a few, amount to little more than lip service so long as our great health services remain outside the purview of the medical student." It is this preventive aspect which I consider most important in games and sports, and of essential importance in the case of those who are physically handicapped or merely shy and backward. It is the doctor's duty to the community to do his best to raise the general health of that community, and in no one way can more be done than by early attention to children and young adults before they have seriously started on their games and sports. Failures and injuries there are bound to be, varying from mild ones, such as mere stiffness and soreness on taking to unaccustomed movements, to serious physical ones, such as strains, pulled muscles and tendons, or from minor mental ones, such as shyness and lack of adaptability and inability to mix normally in sport with other children, to major disturbances such as staleness and even nervous breakdowns in those engaged in intense competitive sport. These fall outside the scope of this article, for they come within the domain of accepted medicine. Excessive stiffness, like strains and pulls and other serious injuries, however, is often due to some unsuspected septic rheumatic focus : shyness and inability to mix, like early staleness or nervous exhaustion, are often due to mild neuroses, whether intrinsic constitutional defects or extrinsic ones due to conflict with environment. A careful physical and mental assessment of every participant would, in a large measure, prevent many of the minor tragedies which are so often seen amongst young people. Prophylaxis is the only satisfactory remedy for ills, mental or physical, in the field of games and sports. Treatment of the fully developed disease is too often mere patching up of damage.

The general practitioner in contact with the very young has the big opportunity of keeping his charges from being ill. He must not become so obsessed with disease and potential disease that he forgets how to deal with healthy individuals as living and thinking human beings.

